# Supplementary material for: Resolving the full spectrum of human genome variation using Linked-Reads
Source: Genome Res. 2019 Apr;29(4):635–45. doi: 10.1101/gr.234443.118 (PMC6442396; doi:10.1101/gr.234443.118)
Supplement: Supplemental Material [file supp_gr.234443.118_Supplemental_longranger-2.2.zip › longranger-2.2.tar.bz2!/lr2.2-public/lib/stan/cmdstan/stan/doc/api/html/structstan_1_1math_1_1promoter.html]

Stan: stan::math::promoter< F, T > Struct Template Reference


|  |  |
| --- | --- |
|  | Stan  2.5.0  probability, sampling & optimization |


- Main Page
- Related Pages
- Namespaces
- Classes
- Files

- Class List
- Class Index
- Class Hierarchy
- Class Members

All Classes Namespaces Files Functions Variables Typedefs Enumerations Enumerator Friends Macros Pages

- stan
- math
- promoter

Static Public Member Functions |
List of all members

stan::math::promoter< F, T > Struct Template Reference

`#include <promoter.hpp>`

|  |  |
| --- | --- |
| Static Public Member Functions | |
| static void | promote (const F &u, T &t) |
|  | |
| static T | promote\_to (const F &u) |
|  | |

## Detailed Description

### template<typename F, typename T> struct stan::math::promoter< F, T >

Definition at line 14 of file promoter.hpp.

## Member Function Documentation

template<typename F, typename T>

|  |  |  |  |
| --- | --- | --- | --- |
| static void stan::math::promoter< F, T >::promote | ( | const F & | *u*, |
|  |  | T & | *t* |
|  | ) |  |  |

inlinestatic

Definition at line 15 of file promoter.hpp.

template<typename F, typename T>

|  |  |  |  |  |  |
| --- | --- | --- | --- | --- | --- |
| static T stan::math::promoter< F, T >::promote\_to | ( | const F & | *u* | ) |  |

inlinestatic

Definition at line 18 of file promoter.hpp.

---

The documentation for this struct was generated from the following file:

- src/stan/math/matrix/promoter.hpp


---

[ Stan Home Page ]

*© 2011–2014,
Stan Development Team.*
